# Pulmonary hyperinflation due to gas trapping and pulmonary artery size: The MESA COPD Study

**DOI:** 10.1371/journal.pone.0176812

**Published:** 2017-05-02

**Authors:** Hooman D. Poor, Steven M. Kawut, Chia-Ying Liu, Benjamin M. Smith, Eric A. Hoffman, João A. Lima, Bharath Ambale-Venkatesh, Erin D. Michos, Martin R. Prince, R. Graham Barr

**Affiliations:** 1 Department of Medicine, Columbia University Medical Center, New York, New York, United States of America; 2 Department of Medicine, Perelman School of Medicine at the University of Pennsylvania, Philadelphia, Pennsylvania, United States of America; 3 Department of Radiology Johns Hopkins University, Baltimore, Maryland, United States of America; 4 Department of Radiology, University of Iowa, Iowa City, Iowa, United States of America; 5 Department of Medicine, Johns Hopkins University, Baltimore, Maryland, United States of America; 6 Department of Radiology, Columbia University Medical Center, New York, New York, United States of America; 7 Department of Epidemiology, Columbia University Medical Center, New York, New York, United States of America; University of California San Francisco, UNITED STATES

## Abstract

**Background:**

Pulmonary hypertension is associated with increased morbidity and mortality in chronic obstructive pulmonary disease (COPD). Since pulmonary artery (PA) size increases in pulmonary hypertension, we measured PA cross-sectional area using magnetic resonance imaging (MRI) to test the hypothesis that pulmonary hyperinflation due to gas trapping is associated with PA cross-sectional area in COPD.

**Methods:**

The MESA COPD Study recruited participants with COPD and controls from two population-based cohort studies ages 50–79 years with 10 or more pack-years and free of clinical cardiovascular disease. Body plethysmography was performed according to standard criteria. Cardiac MRI was performed at functional residual capacity to measure the cross-sectional area of the main PA. Percent emphysema was defined as the percentage of lung voxels less than -950 Hounsfield units as assessed via x-ray computed tomography. Analyses were adjusted for age, gender, height, weight, race-ethnicity, the forced expiratory volume in one second, smoking status, pack-years, lung function, oxygen saturation, blood pressure, left ventricular ejection fraction and percent emphysema.

**Results:**

Among 106 participants, mean residual volume was 1.98±0.71 L and the mean PA cross-sectional area was 7.23±1.72 cm^2^. A one standard deviation increase in residual volume was independently associated with an increase in main PA cross-sectional area of 0.55 cm^2^ (95% CI 0.18 to 0.92; p = 0.003). In contrast, there was no evidence for an association with percent emphysema or total lung capacity.

**Conclusion:**

Increased residual volume was associated with a larger PA in COPD, suggesting that gas trapping may contribute to pulmonary hypertension in COPD.

## Introduction

Chronic obstructive pulmonary disease (COPD) is characterized by accelerated age-related loss in lung function and progressive airflow obstruction that is only partially reversible [1]. COPD is the third leading cause of mortality in the United States [2]. COPD may be complicated by the development of pulmonary hypertension (PH), although the severity of PH in COPD patients is usually mild [3]. The presence of PH in COPD is associated with increased mortality [4], increased hospitalizations [5], and reduced exercise capacity [6, 7].

The gold standard to diagnose and characterize the degree of PH is right heart catheterization, a relatively safe but still invasive procedure. Echocardiographic estimates of pulmonary artery (PA) pressures are subject to significant error in patients with COPD, given the technical challenges that arise from pulmonary hyperinflation with hyperexpansion of the chest [8, 9]. PA dilatation is a consequence of PH and its presence can be used to infer PH noninvasively with various imaging modalities, including cardiac magnetic resonance imaging (MRI) [10–13]. In addition, an increased ratio of PA:aortic diameter as measured on computed tomography (CT) was associated with severe exacerbations of COPD [14].

PH in COPD is caused by a combination of factors, including alveolar hypoxia acutely causing hypoxic pulmonary vasoconstriction [15], chronically contributing to pulmonary vascular remodeling [16, 17], hypercapnia and acidosis [18, 19], along with comorbidities including obstructive sleep apnea, left ventricular systolic or diastolic dysfunction and thromboembolic disease [20]. Although increases in PA pressure can be provoked by gas trapping and lung hyperinflation during hyperventilation [21], exercise [22] and acute exacerbations [23], it is unclear whether gas trapping and hyperinflation contribute to PH in stable COPD patients. Destruction of the pulmonary vascular bed has also been hypothesized as contributing to PH in emphysema, however; there is a lack of substantial evidence to support this mechanism [20]. In the classic literature, emphysematous COPD was not characterized by PH [24] and, in a recent report from this cohort, emphysematous COPD was associated with smaller not larger right ventricular volumes [25]. Recent studies have linked early pathology associated with smoking associated emphysema to both loss of peripheral airways [26] and heterogeneous parenchymal perfusion [27].

We hypothesized that greater residual volume, a standard clinical measure of pulmonary hyperinflation and alveolar gas trapping on body plethysmography [28], would be independently associated with larger PA cross-sectional area as measured by cardiac MRI. In addition, to assess a possible confounding factor of parenchymal destruction, we also examined if the degree of emphysema-like lung (percent emphysema) on computed tomography (CT) and thoracic hyperexpansion were independently associated with PA cross-sectional area.

## Materials and methods

### Study participants

The MESA COPD Study is a cross-sectional study of smokers nested among the Multi-Ethnic Study of Atherosclerosis (MESA)[29] and the Emphysema and Cancer Action Project (EMCAP)[30]. Inclusion criteria were age 50–79 years old and ≥10 pack-year smoking history. Exclusion criteria included clinical cardiovascular disease (heart failure, valve disease, coronary artery disease, stroke, congenital heart disease or current atrial fibrillation), asthma prior to age 45 years, pulmonary embolism and other lung diseases, weight > 300 lbs, Stage III/IV chronic kidney disease insufficiency, and contraindications to MRI, gadolinium, albuterol or spirometry. This report includes the 106 participants who completed chest CT, body plethysmography and cardiac MRI.

### Pulmonary function testing

A V6200 Series Autobox (Sensormedics, Yorba Linda, CA) and an OMI rolling barrel spirometer were used to perform body plethysmography and post-bronchodilator spirometry, respectively, following American Thoracic Society/European Respiratory Society (ATS/ERS) recommendations [31, 32] and as has been previously described [33]. Functional residual capacity (FRC) was measured while panting at 0.5 to 1 breaths per second. At least two technically satisfactory maneuvers were performed, followed by an inspiratory capacity and slow vital capacity maneuver. The mean of the satisfactory measurements was used for the FRC. The total lung capacity (TLC) was calculated as the sum of the FRC and the inspiratory capacity (IC). The residual volume was calculated as the difference between the TLC and the slow vital capacity. Hankinson reference equations were used to calculate predicted spirometry values [33]. ATS/ERS COPD criteria were used to assess COPD status and severity [34]. Predicted lung volume values and upper and lower limits of normal for each lung volume were calculated using Garcia-Rio reference equations for participants 65 years and older, and Crapo reference equations for participants under 65 years [35, 36].

Gas trapping was defined as residual volume or residual volume/TLC above the upper limit of normal; hyperexpansion was defined as FRC or TLC above the upper limit of normal.[37]

### Cardiac MRI and pulmonary artery cross-sectional area analysis

Cardiac MRI was performed at 1.5 Tesla (Signal LX, GE Healthcare, Waukesha, WI) using the MESA protocol as previously described [29]. An oblique localizer prescribed from axial and coronal images was used to visualize the natural curvature of the pulmonary artery. This oblique localizer was used to prescribe a plane perpendicular to the main PA positioned above the pulmonary valve but below the pulmonary bifurcation. The right PA was imaged between the trachea and the ascending aorta using a coronal localizer. A localizer oblique to the plane of the left PA was prescribed from an axial image and used for obtaining the plane perpendicular to the left PA. For main, right and left PA, phase-contrast images were obtained using a segmented gradient echo sequence (TR/TE = 7.5/3.1 msec, slice thickness 8 mm, matrix 256x256, 2 views/segment, 30 cardiac phases over the cardiac cycle, two averages, velocity encoding 100 cm/s) without breath holding. For each PA, the maximum/minimum diameter and cross-sectional areas were measured with electronic calibers (Advantage Windows Workstation, GE Healthcare, Waukesha, WI). The coefficient of variation for the inter-reader variability of the main PA measures was 6%. The maximum and minimum areas obtained of the PA during the cardiac cycle were deemed the systolic and diastolic areas respectively.

To measure left ventricular function, the entire heart was imaged in short-axis orientation with 12 or more slices. All cine images had temporal resolution of 48 msec and were retrospectively reconstructed at overlapping 20–35 msec intervals over the cardiac cycle with 40 phases. Semi-automated contouring was used to determine left ventricular volumes and ejection fraction (Cardiac Image Modeller, New Zealand) [38]. Cardiac MR readers were blinded from other participant information.

To measure the volume of the left atrium (LA), an experienced operator defined the LA endocardial and epicardial borders at the frame of the largest LA volume using the Multimodality Tissue tracking software (MTT; version 6.0, Toshiba, Japan) on long-axis 2-chamber and 4-chamber cine MR images. The volume was calculated using the biplane method as follows: LA volume = (0.848 * area4chamber * area2chamber)/(length2chamber + length4chamber)/2 [39].

### Assessment of percent emphysema on CT

A GE 64-slice helical scanner (120 kVp, 200mAs at 0.5 seconds) with 0.75 mm slice thickness was used to perform full-lung thoracic CT on the participants. Images were obtained at suspended full inspiration. Percent emphysema was assessed via the Apollo software (VIDA Diagnostics, Coralville IA) at a single, independent reading center blinded to other participant The percentage of total voxels within the lung field with values below -950 Hounsfield units (HU) was designated as percent emphysema (also known as percent low attenuation areas) [40].

*Anthropometry*, *blood pressure and other co-variates*: Age, gender and race or ethnic group were self-reported. Height, weight, seated blood pressure were measured according to the MESA protocol [41]. Smoking history was self-reported using a standard questionnaire and smoking status was confirmed with plasma and urine cotinine levels (Immulite 2000 Nicotine Metabolite Assay; Diagnostic Products Corp., Los Angeles, CA, USA). Oxygen saturation was measured non-invasively at rest with pulse oximetry. Serum bicarbonate was measured on clinical chemistries.

### Study oversight

The institutional review boards of the participating institutions, which include Columbia University, UCLA, Johns Hopkins, University of Iowa, Northwestern University, University of Washington, and the National Heart, Lung, and Blood Institute, approved the study procedures. Written informed consent was obtained from all participants in the MESA COPD Study.

### Statistical analysis

The cohort was stratified by quartiles of percent predicted residual volume for descriptive purposes. The initial analysis of the relationship between systolic and diastolic PA cross-sectional area and residual volume was performed using multiple linear regression with adjustment for the following potential confounders and precision variables: age, gender, height, weight, race-ethnicity, FEV1, resting oxygen saturation, cohort and percent emphysema. The full model additionally included smoking history and status, blood pressure and left ventricular ejection fraction. To minimize the possibility of confounding by body size, the analyses were repeated for another measure of hyperinflation, residual volume to TLC ratio (RV/TLC). Analyses for PA cross-sectional area and percent emphysema proceeded in a similar fashion. Analyses were weighted according to the inverse ratio of sample prevalence to source study prevalence, as previously described [37], to avoid bias for the recruitment based on COPD status. To account for multiple comparisons of the two primary endpoints, systolic and diastolic cross-sectional areas of the main PA, we used a two-tailed, Bonferroni-corrected P-value of 0.025 to define statistical significance. If significant, we additionally performed secondary analyses of the right and left PA cross-sectional areas.

## Results

Of the 106 participants, the mean age was 69.5±5 years and 57% were male. Table 1 summarizes the characteristics of the study participants stratified by quartile of percent predicted residual volume. Sixty-five percent of the participants had COPD, mostly of mild or moderate severity. The proportion of males and current smokers increased across categories of residual volume, as did severity of COPD, alternate measures of hyperinflation and percent emphysema. Height and weight increased across categories of residual volume but BMI did not; oxygen saturation and bicarbonate were similar across categories, and left ventricular ejection fraction was marginally lower only in the category with the greatest residual volume. Twenty-seven participants (25%) had a residual volume greater than 100% predicted and 18 participants (17%) had a residual volume greater than 110% predicted.

**Table 1:** Baseline characteristics of MESA COPD Study participants with plethysmograph and MRI measures*. Stratified by quartiles percent predicted residual volume.

*Characteristic*	*Quartiles of Percent Predicted Residual Volume*			
	1	2	3	4
% Predicted Residual Volume—median No.	59% N = 26	77% N = 27	90% N = 26	120% N = 27
Age—years	70±4	71±5	71±5	67±6
Male sex—no. (%)	9 (35)	13 (48)	16 (62)	19 (70)
Race or ethnic group—no. (%)†				
Caucasian	17 (65)	23 (85)	21 (81)	20 (74)
African American	4 (15)	2 (7)	3 (12)	7 (26)
Other	5 (19)	2 (7)	2 (8)	0 (0)
Height—cm	166±10	167±10	169±8	171±10
Weight—kg	75±17	77±15	76±18	78±21
BMI—kg/m^2^	27±6	27±5	27±5	26±5
Cigarette smoking status—no. (%)†				
Former smoker	19 (73)	18 (67)	17 (65)	13 (48)
Current smoker	7 (27)	9 (33)	9 (35)	14 (52)
Pack-years of smoking—no.†				
Median (IQR)	36 (32)	40 (25)	43 (30)	51 (34)
Serum bicarbonate—mEq/L	27 (1)	27 (2)	27 (3)	28 (3)
Blood pressure—mm Hg				
Systolic	119±18	123±15	125±18	122±14
Diastolic	67±10	69±11	73±11	74±8
COPD—no. (%)	10 (38)	13 (48)	17 (65)	25 (93)
GOLD—COPD severity—no. (%)				
Mild	5 (19)	8 (30)	8 (31)	3 (11)
Moderate	5 (19)	5 (19)	8 (31)	13 (48)
Severe / very severe	0 (0)	0 (0)	1 (4)	9 (33)
Plethysmography:				
Residual volume—L	1.24±0.23	1.69±0.16	2.08±0.23	2.89±0.66
FRC—L	2.81±0.78	2.96±0.58	3.38±0.56	4.15±0.73
% Predicted FRC	89.9±18.9	93.4±16.0	104.3±15.5	125.2±19.0
TLC—L	4.82±1.07	5.42±1.11	5.84±1.09	6.34±1.12
% Predicted TLC	85.9±11.9	93.1±10.4	96.9±9.1	102.3±12.7
Residual volume to TLC ratio	0.26±0.05	0.32±0.05	0.37±0.06	0.46±0.08
Post-bronchodilator spirometry:				
FEV_1_—L	2.32±0.60	2.41±0.70	2.31±0.78	1.72±0.67
% Predicted FEV_1_	95.8±15.7	94.0±20.4	88.1±25.2	60.4±18.5
FVC—L	3.27±0.82	3.56±0.92	3.57±1.01	3.37±0.93
% Predicted FVC	101.8±14.9	103.9±17.8	100.7±17.9	88.1±17.9
FEV_1_ to FVC ratio	0.71 ±0.08	0.68±0.10	0.64±0.11	0.51±0.10
Diffusion capacity:				
DLCO—mL/min/mmHg	16.62±4.79	16.99±5.28	16.84±4.41	15.54±6.11
%Predicted DLCO	64.0±10.6	63.2±12.6	61.7±13.8	52.7±17.9
Percent emphysema_-950 HU_—median (IQR)	1.1 (2.5)	1.3 (2.6)	1.8 (2.0)	7.89 (13)
Oxygen saturation—median (IQR)	97 (3)	97 (3)	97 (3)	97 (2)
Left ventricular ejection fraction—%	61±6	61±7	63±8	59±6
Left atrial volume—mL	59.7±28.7	62.1±21.6	57.4±22.7	62.9±21.1

* Plus-minus values are means ± standard deviation.

^†^Race or ethnic group, smoking status and pack-year history were self-reported.

Abbreviations: COPD denotes chronic obstructive pulmonary disease, FRC functional residual capacity, TLC total lung capacity, FEV_1_ forced expired volume in the first second, FVC forced vital capacity, and HU Hounsfield units.

### Pulmonary hyperinflation/gas trapping and PA dimension

Both systolic and diastolic main PA cross-sectional areas rose monotonically across categories of residual volume (Table 2). A significant association was observed between residual volume, and the systolic cross-sectional area of the main PA. In the fully adjusted model, a one standard deviation increase in residual volume (0.71 L) was associated with a 0.55 cm^2^ increase in the main PA systolic cross-sectional area (95% CI 0.18 to 0.92; p = 0.003). Results were very similar and equally significant for the main PA diastolic cross-sectional area (Table 2). We additionally examined the right and left PA: there were similar significant associations between residual volume and the right PA cross-sectional area but no association between residual volume and the left PA cross-sectional area.

**Table 2:** Relationship between residual volume and pulmonary artery cross-sectional area*.

*Pulmonary Artery*	*Mean difference in quartiles of Residual Volume*				*Mean difference in pulmonary artery cross-sectional area per standard deviation increase in RV (95% CI)* *n = 106*	*P Value*
% Predicted Residual Volume—median	59%	77%	90%	120%		
Main Pulmonary Artery Systolic—cm^2^	6.6±1.6	7.1±1.2	7.4±2.2	7.8±1.7		
Model 1	0	0.33	0.53	0.64	0.43 (-0.01 to 0.86)	0.055
Model 2	0	0.38	0.85	0.82	0.55 (0.18 to 0.92)	0.003
Main Pulmonary Artery Diastolic—cm^2^	5.8±1.4	6.2±1.1	6.5±2.0	7.0±1.6		
Model 1	0	0.37	0.45	0.56	0.38 (-0.01 to 0.78)	0.059
Model 2	0	0.41	0.75	0.71	0.51 (0.12 to 0.90)	0.01
Right Pulmonary Artery Systolic—cm^2^	3.8±1.0	4.7±0.8	4.7±1.2	5.1±1.4		
Model 1	0	1.20	0.96	1.83	0.63 (0.16 to 1.11)	0.009
Model 2	0	1.22	1.00	1.82	0.66 (0.21 to 1.12)	0.004
Right Pulmonary Artery Diastolic—cm^2^	3.2±0.8	4.0±0.8	3.9±1.0	4.3±1.2		
Model 1	0	1.03	0.77	1.60	0.55 (0.12 to 0.98)	0.01
Model 2	0	1.02	0.75	1.56	0.56 (0.16 to 0.97)	0.006
Left Pulmonary Artery Systolic—cm^2^	4.1±1.0	4.1±1.2	4.2±1.3	4.8±1.5		
Model 1	0	0.30	-0.30	0.18	-0.24 (-0.73 to 0.25)	0.34
Model 2	0	0.25	-0.54	0.13	-0.30 (-0.76 to 0.15)	0.19
Left Pulmonary Artery Diastolic—cm^2^	3.6±1.0	3.5±1.0	3.6±1.1	4.1±1.3		
Model 1	0	0.15	-0.38	0.05	-0.26 (-0.70 to 0.18)	0.25
Model 2	0	0.10	-0.60	-0.01	-0.31 (-0.70 to 0.08)	0.12

*Mean differences in model 1 were adjusted for age, gender, race or ethnic group, height, weight, cohort, percent emphysema_-950 HU_, forced expired volume in the first second and oxygen saturation. Model 2 was additionally adjusted for smoking status, pack-years of smoking history, systolic blood pressure, diastolic blood pressure and left ventricular ejection fraction. Plus-minus values are means ± standard deviation. Abbreviations: CI denotes confidence interval, HU Hounsfield units, RV residual volume and FEV_1_ forced expired volume in the first second.

Similar associations of often greater statistical significance were observed between RV/TLC and PA cross-sectional areas (S1 Table).

**Fig 1:**
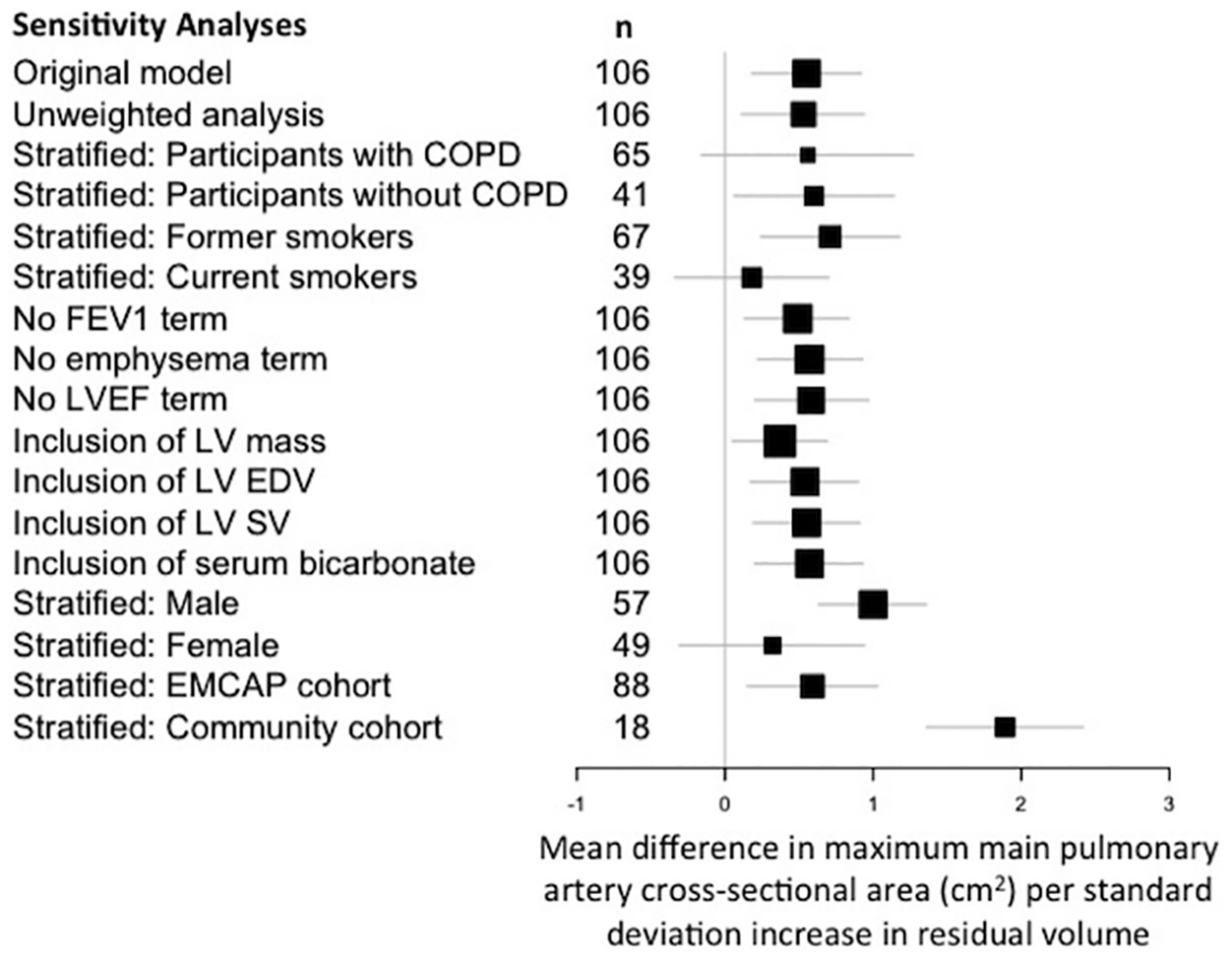
Mean difference in systolic main pulmonary artery cross-sectional area by residual volume. Original model was adjusted for age, gender, race or ethnic group, height, weight, cohort, cohort, percent emphysema_-950 HU_, forced expired volume in the first second and oxygen saturation, smoking status, pack-years of smoking history, systolic blood pressure, diastolic blood pressure and left ventricular ejection fraction.

Additional sensitivity analyses revealed no significant interaction between these associations and gender, smoking status, cohort, presence of COPD, serum bicarbonate, left ventricular mass, left ventricular stroke volume, left ventricular end-diastolic volume. (Fig 1). When stratifying the results for with COPD, the point estimate for the effect of RV on PA cross-sectional area did not change but the confidence intervals widened, likely indicating that the study was underpowered for this subgroup and not that there was an effect modification of the relationship by COPD status.

### Percent emphysema and hyperexpansion and PA dimensions

In contrast to the results for pulmonary hyperinflation and gas trapping, there was no evidence for an association between percent emphysema and any PA cross-sectional area (Table 3). There was also no evidence for an association between PA cross-sectional areas and other measures of hyperinflation suggestive of hyperexpansion, including TLC and FRC.

**Table 3:** Relationship between percent emphysema and pulmonary artery cross-sectional area*.

*Pulmonary Artery*	*Mean difference in pulmonary artery cross-sectional area per standard deviation increase in log-transform percent emphysema (95% CI)*	*P Value*
Main Pulmonary Artery Systolic—cm^2^		
Model 1	0.02 (-0.23 to 0.27)	0.85
Model 2	0.02 (-0.24 to 0.28)	0.88
Main Pulmonary Artery Diastolic—cm^2^		
Model 1	0.06 (-0.17 to 0.29)	0.60
Model 2	0.08 (-0.15 to 0.31)	0.49
Right Pulmonary Artery Systolic—cm^2^		
Model 1	-0.01 (-0.18 to 0.15)	0.88
Model 2	0.002 (-0.16 to 0.17)	0.98
Right Pulmonary Artery Diastolic—cm^2^		
Model 1	0.02 (-0.12 to 0.17)	0.75
Model 2	0.05 (-0.91 to 0.19)	0.50
Left Pulmonary Artery Systolic—cm^2^		
Model 1	0.08 (-0.10 to 0.27)	0.38
Model 2	0.11 (-0.07 to 0.29)	0.24
Left Pulmonary Artery Diastolic—cm^2^		
Model 1	0.09 (-0.08 to 0.25)	0.31
Model 2	0.12 (-0.05 to 0.28)	0.16

*Mean differences in model 1 were adjusted for age, gender, race or ethnic group, height, weight, cohort, forced expired volume in the first second and oxygen saturation. Model 2 was additionally adjusted for smoking status, pack-years of smoking history, systolic blood pressure, diastolic blood pressure and left ventricular ejection fraction. Abbreviations: CI denotes confidence interval.

### Right ventricular measures and PA dimensions

There was no evidence for an association between right ventricular parameters and main PA cross-sectional area. (S2 and S3 Tables)

## Discussion

Pulmonary hyperinflation and specifically gas trapping, as assessed by increased residual volume and residual volume to TLC ratio, was associated with a larger main PA cross-sectional area on MRI. In contrast, there was no evidence that the extent of emphysema as measured by CT or measures of hyperexpansion (increased FRC and TLC) were associated with PA dimensions. These findings suggest that gas trapping, but not emphysema, are associated with pulmonary vascular remodeling in current and former smokers.

The present report is the first, to the best of our knowledge, to consider the relationship between pulmonary hyperinflation, gas trapping and PA size. A few prior studies have shown a relationship between pulmonary hyperinflation and increased PA pressure. The classic experiments by West elucidated the relationship between alveolar pressure and regional pulmonary blood flow, demonstrating that increasing alveolar pressure in the presence of constant pulmonary arterial pressure results in decreasing regional pulmonary blood flow. These experiments helped define the concept of the “West lung zones” [42]. Hakim and colleagues, using a canine model, determined that pulmonary vascular resistance increases with lung inflation, both via positive-pressure and negative-pressure breathing, suggesting that alveolar vessels behave as Starling resistors [43]. Fuld et al., utilizing both dynamic computed tomography to directly assess pulmonary perfusion and dual energy CT to assess regional pulmonary blood volume, demonstrated the role of increasing lung volume in limiting both perfusion and regional blood volume in the non-dependent lung regions with the extent of the effect increasing with the level of lung inflation [44]. The association between increased PA cross-sectional area and hyperinflation with gas trapping found in our study may be a result of the compression of alveolar vessels from increased alveolar pressure and volume, leading to an increase in pulmonary vascular resistance and consequently an increase in PA pressure. While a similar association between PA pressure and hyperinflation with gas trapping was found for the right PA, there was no association for the left PA. This discrepancy may have occurred because the right PA is less constrained by mediastinal structures than the left PA, possibly making its cross-sectional area a better gauge of intravascular pressure.

The effect of gas trapping and hyperinflation on PA pressure and pulmonary vascular resistance in patients with COPD has been previously studied in small but interventional studies. Harris and colleagues reported that voluntary hyperventilation to achieve high minute ventilation in patients with chronic bronchitis resulted in increased PA pressures and increased pulmonary vascular resistance but that it had no such effect in normal patients [45]. Similarly, Butler and colleagues found an increase in PA pressure and pulmonary capillary wedge pressure with hyperventilation in patients with COPD but not in normal patients [21]. In a recent study examining explanted lungs from 17 patients with severe COPD, half of them with mild pulmonary hypertension, pulmonary vascular involvement was unexpectedly modest, indicating a mechanism other than a primary vasculopathy as the cause of pulmonary hypertension [46]. These studies suggest that dynamic hyperinflation may be the major contributor to hemodynamic changes.

It has been postulated that emphysematous destruction of the pulmonary vasculature contributes to elevated pulmonary vascular pressures in COPD [47]. However, our study found no association between the extent of emphysema as measured quantitatively on CT and PA cross-sectional area. Our finding is consistent with prior studies examining the relationship between PA pressures and the extent of emphysema. Scharf and colleagues found no association between mean PA pressures and CT emphysema scores in the 120 patients with severe emphysema from the National Emphysema Treatment trial [48]. Biernacki and colleagues found no association between mean PA pressures, both at rest and with exercise, and CT measures of emphysema in 32 patients with COPD.[49] Burrows and colleagues, in their classic paper examining the different hemodynamic phenotypes in COPD, noted that patients with a predominantly “emphysematous” type of COPD often had PA pressures within normal limits and only slight elevations in pulmonary vascular resistance [24]. Furthermore, we recently reported that COPD and particularly emphysema was associated, on average, with smaller right ventricular volumes and that this was particularly true for emphysema [25]. Of note, the association of gas trapping with increased PA dimensions reported here did not translate into right ventricular enlargement, possibly related to the generally mild-moderate severity of gas trapping in this cohort.

The present findings and above studies suggest that impaired left ventricular filling and concomitant reductions in stroke volume related to percent emphysema, which have been previously observed in a large, population-based cohort free of clinical cardiovascular disease [50] and in the present cohort [51], may be related to vascular destruction in emphysema that reduces pulmonary blood flow but which does not markedly increase PA pressure.

The present study has several limitations. First, because pulmonary cross-sectional area was measured rather than PA pressure, the proposed mechanism of increased PA pressure from downstream alveolar vessel compression leading to PA dilatation remains speculative. Conceivably, hyperinflation could cause architectural changes of the pulmonary vasculature leading to an increase in PA cross-sectional area without necessarily an increase in PA pressure. The testing necessary to demonstrate whether the increased PA cross-sectional area is from increased PA pressure or another cause are too invasive to be applied to a population-based study of participants free of clinical cardiovascular disease with predominantly mild to moderate COPD. Further work is needed to elucidate the direct relationship between hyperinflation and PA pressure by directly measuring or estimating PA pressures in patients with mild to moderate COPD. Second, residual confounding by left ventricular diastolic dysfunction may have contributed to the observed association. Smith and colleagues, in the same cohort as this study, observed that greater residual volume and RV/TLC ratio is associated with greater left ventricular mass [37]. However, in our study, sensitivity analyses revealed no effect when adjusting for left ventricular end-diastolic mass, left ventricular end-diastolic volume and left ventricular stroke volume. Third, we did not perform arterial blood gasses; however, there was no suggestion that hypoxemia and hypercapnea varied substantially with residual volume in this cohort and sensitivity analyses revealed no effect when adjusting for serum bicarbonate. Fourth, the cross-sectional design of the study does not allow for determination of cause and effect for the observed association; however, it seems much more likely from a physiologic standpoint that hyperinflation would cause increased PA cross-sectional area as opposed to the other way around.

## Conclusions

In conclusion, increased residual volume, a measure of pulmonary hyperinflation and gas trapping, was independently associated with increased PA cross-sectional area. In contrast, there was no evidence of a relationship of emphysema severity to PA dimensions. These results suggest that gas trapping may be a cause of increased PA pressure in COPD, possibly from alveolar vessel compression by distended alveoli.

## Supporting information

S1 Table Relationship between residual volume–Total lung capacity ratio and pulmonary artery cross-sectional area. Mean differences in model 1 were adjusted for age, gender, race or ethnic group, height, weight, cohort, percent emphysema_-950 HU_, forced expired volume in the first second and oxygen saturation. Model 2 was additionally adjusted for smoking status, pack-years of smoking history, systolic blood pressure, diastolic blood pressure and left ventricular ejection fraction. Abbreviations: CI denotes confidence interval, HU Hounsfield units, RV residual volume, TLC total lung capacity and FEV_1_ forced expired volume in the first second.
(DOCX)

S2 Table Relationship between right ventricle parameters and main pulmonary artery systolic cross-sectional area. Models were adjusted for age, gender, race or ethnic group, height, weight, cohort, percent emphysema_-950 HU_, forced expired volume in the first second, oxygen saturation, smoking status, pack-years of smoking history, systolic blood pressure, and diastolic blood pressure. Abbreviations: CI denotes confidence interval, HU Hounsfield units, RV right ventricle.
(DOCX)

S3 Table Relationship between right ventricle parameters and main pulmonary artery diastolic cross-sectional area. Models were adjusted for age, gender, race or ethnic group, height, weight, cohort, percent emphysema_-950 HU_, forced expired volume in the first second, oxygen saturation, smoking status, pack-years of smoking history, systolic blood pressure, and diastolic blood pressure. Abbreviations: CI denotes confidence interval, HU Hounsfield units, RV right ventricle.
(DOCX)
