# Supplementary material for: Pulmonary hyperinflation due to gas trapping and pulmonary artery size: The MESA COPD Study
Source: PLoS One. 2017 May 2;12(5):e0176812. doi: 10.1371/journal.pone.0176812 (PMC5413010; doi:10.1371/journal.pone.0176812)
Supplement: S1 Table — Mean differences in model 1 were adjusted for age, gender, race or ethnic group, height, weight, cohort, percent emphysema-950 HU, forced expired volume in the first second and oxygen saturation. Model 2 was additionally adjusted for smoking status, pack-years of smoking history, systolic blood pressure, diastolic blood pressure and left ventricular ejection fraction. Abbreviations: CI denotes confidence interval, HU Hounsfield units, RV residual volume, TLC total lung capacity and FEV1 forced expired volume in the first second. (DOCX) [file pone.0176812.s001.docx]

**Online Supplement Table 1. Relationship Between Residual Volume – Total Lung**

**Capacity Ratio and Pulmonary Artery Cross-sectional Area***

| *Pulmonary Artery* | | *Mean difference in quartiles of*  *RV/TLC* | | | | *Mean difference in pulmonary artery cross-sectional area per standard deviation increase*  *in RV (95% CI)*  *n = 106* | | *P Value* |
| --- | --- | --- | --- | --- | --- | --- | --- | --- |
| RV/TLC – median | 0.26 | | 0.31 | 0.37 | 0.47 | |  |  |
| Main Pulmonary Artery Systolic – cm^2^ |  | |  |  |  | |  |  |
| Model 1 | 0 | | 0.31 | 0.10 | 1.33 | | 0.44 (0.04 to 0.85) | 0.03 |
| Model 2 | 0 | | 0.14 | 0.26 | 1.46 | | 0.53 (0.16 to 0.90) | 0.005 |
| Main Pulmonary Artery Diastolic – cm^2^ |  | |  |  |  | |  |  |
| Model 1 | 0 | | 0.30 | 0.06 | 1.01 | | 0.36 (-0.01 to 0.73) | 0.056 |
| Model 2 | 0 | | 0.16 | 0.24 | 1.15 | | 0.51 (0.10 to 0.91) | 0.01 |
| Right Pulmonary Artery Systolic – cm^2^ |  | |  |  |  | |  |  |
| Model 1 | 0 | | 0.47 | 0.73 | 1.61 | | 0.67 (0.22 to 1.1) | 0.003 |
| Model 2 | 0 | | 0.53 | 0.94 | 1.71 | | 0.78 (0.35 to 1.2) | <0.001 |
| Right Pulmonary Artery Diastolic – cm^2^ |  | |  |  |  | |  |  |
| Model 1 | 0 | | 0.45 | 0.66 | 1.31 | | 0.56 (0.16 to 0.96) | 0.006 |
| Model 2 | 0 | | 0.51 | 0.81 | 1.42 | | 0.64 (0.27 to 1.0) | <0.001 |
| Left Pulmonary Artery Systolic – cm^2^ |  | |  |  |  | |  |  |
| Model 1 | 0 | | -0.23 | -0.52 | -0.44 | | -0.27 (-0.73 to 0.19) | 0.25 |
| Model 2 | 0 | | -0.03 | -0.71 | -0.59 | | -0.30 (-0.75 to 0.15) | 0.19 |
| Left Pulmonary Artery Diastolic – cm^2^ |  | |  |  |  | |  |  |
| Model 1 | 0 | | -0.33 | -0.60 | -0.63 | | -0.33 (-0.73 to 0.08) | 0.11 |
| Model 2 | 0 | | -0.14 | -0.75 | -0.75 | | -0.35 (-0.74 to 0.04) | 0.08 |

*Mean differences in model 1 were adjusted for age, gender, race or ethnic group, height, weight, cohort, percent emphysema_-950 HU_, forced expired volume in the first second and oxygen saturation. Model 2 was additionally adjusted for smoking status, pack-years of smoking history, systolic blood pressure, diastolic blood pressure and left ventricular ejection fraction. Abbreviations: CI denotes confidence interval, HU Hounsfield units, RV residual volume, TLC total lung capacity and FEV_1_ forced expired volume in the first second.
