# Supplementary material for: Pulmonary hyperinflation due to gas trapping and pulmonary artery size: The MESA COPD Study
Source: PLoS One. 2017 May 2;12(5):e0176812. doi: 10.1371/journal.pone.0176812 (PMC5413010; doi:10.1371/journal.pone.0176812)
Supplement: S2 Table — Models were adjusted for age, gender, race or ethnic group, height, weight, cohort, percent emphysema-950 HU, forced expired volume in the first second, oxygen saturation, smoking status, pack-years of smoking history, systolic blood pressure, and diastolic blood pressure. Abbreviations: CI denotes confidence interval, HU Hounsfield units, RV right ventricle. (DOCX) [file pone.0176812.s002.docx]

**Online Supplement Table 2. Relationship Between Right Ventricle Parameters and Main Pulmonary Artery Systolic Cross-Sectional Area***

| *Right Ventricle Parameters* | | *Mean difference in quartiles of main pulmonary artery systolic cross-sectional area* | | | | *Mean difference per standard deviation increase in main pulmonary artery systolic cross-sectional area (95% CI)*  *n = 106* | | *P Value* |
| --- | --- | --- | --- | --- | --- | --- | --- | --- |
| Quartiles of Main Pulmonary Artery  Systolic Cross-Sectional Area – cm^2^ | 6.2 | | 7.1 | 8.2 | 13.2 | |  |  |
| RV End-Diastolic Volume – mL | 0 | | -0.31 | 9.38 | 1.21 | | 1.79 (-3.4 to 6.99) | 0.50 |
| RV End-Systolic Volume – mL | 0 | | 1.63 | 9.28 | 3.88 | | 2.14 (-1.54 to 5.82) | 0.26 |
| RV Stroke Volume – mL | 0 | | -1.94 | 0.10 | -2.66 | | -0.35 (-3.32 to 2.62) | 0.82 |
| RV End-Diastolic Mass – g | 0 | | 0.41 | 0.23 | -0.86 | | 0.20 (-12 to 1.6) | 0.78 |
| RV End-Diastolic Mass/RV End-Diastolic Volume Ratio – g/mL | 0 | | 0.001 | -0.01 | -0.02 | | -0.003 (-0.01 to 0.01) | 0.59 |
| RV Ejection Fraction – % | 0 | | -2.3 | -4.4 | -3.1 | | -1.01 (-2.62 to 0.61) | 0.22 |

*Models were adjusted for age, gender, race or ethnic group, height, weight, cohort, percent emphysema_-950 HU_, forced expired volume in the first second, oxygen saturation, smoking status, pack-years of smoking history, systolic blood pressure, and diastolic blood pressure.

Abbreviations: CI denotes confidence interval, HU Hounsfield units, RV right ventricle.
