# Supplementary material for: Pulmonary hyperinflation due to gas trapping and pulmonary artery size: The MESA COPD Study
Source: PLoS One. 2017 May 2;12(5):e0176812. doi: 10.1371/journal.pone.0176812 (PMC5413010; doi:10.1371/journal.pone.0176812)
Supplement: S3 Table — Models were adjusted for age, gender, race or ethnic group, height, weight, cohort, percent emphysema-950 HU, forced expired volume in the first second, oxygen saturation, smoking status, pack-years of smoking history, systolic blood pressure, and diastolic blood pressure. Abbreviations: CI denotes confidence interval, HU Hounsfield units, RV right ventricle. (DOCX) [file pone.0176812.s003.docx]

**Online Supplement Table 3. Relationship Between Right Ventricle Parameters and Main Pulmonary Artery Diastolic Cross-Sectional Area***

| *Right Ventricle Parameters* | | *Mean difference in quartiles of main pulmonary artery diastolic cross-sectional area* | | | | *Mean difference per standard deviation increase in main pulmonary artery diastolic cross-sectional area (95% CI)*  *n = 106* | | *P Value* |
| --- | --- | --- | --- | --- | --- | --- | --- | --- |
| Quartiles of Main Pulmonary Artery Diastolic Cross-Sectional Area – cm^2^ | 5.4 | | 6.2 | 7.2 | 12.7 | |  |  |
| RV End-Diastolic Volume – mL | 0 | | 0.97 | 5.98 | 1.87 | | 1.00 (-4.29 to 6.29) | 0.71 |
| RV End-Systolic Volume – mL | 0 | | 0.65 | 7.43 | 1.55 | | 1.77 (-1.82 to 0.36) | 0.33 |
| RV Stroke Volume – mL | 0 | | 0.32 | -1.46 | 0.32 | | -0.77 (-3.92 to 2.38) | 0.63 |
| RV End-Diastolic Mass – g | 0 | | -0.44 | 0.57 | -1.75 | | 0.23 (-1.35 to 1.80) | 0.78 |
| RV End-Diastolic Mass/RV End-Diastolic Volume Ratio – g/mL | 0 | | -0.01 | -0.01 | -0.02 | | -0.002 (-0.01 to 0.01) | 0.70 |
| RV Ejection Fraction – % | 0 | | -1.0 | -4.2 | -1.0 | | -1.10 (-2.72 to 0.52) | 0.18 |

*Models were adjusted for age, gender, race or ethnic group, height, weight, cohort, percent emphysema_-950 HU_, forced expired volume in the first second, oxygen saturation, smoking status, pack-years of smoking history, systolic blood pressure, and diastolic blood pressure.

Abbreviations: CI denotes confidence interval, HU Hounsfield units, RV right ventricle.
